# The Effects of Bariatric Surgery on Cardiovascular Outcomes and Cardiovascular Mortality: A Systematic Review and Meta-Analysis

**DOI:** 10.7759/cureus.34723

**Published:** 2023-02-07

**Authors:** Harshith Chandrakumar, Nazima Khatun, Tanuj Gupta, Suzette Graham-Hill, Angelina Zhyvotovska, Samy I McFarlane

**Affiliations:** 1 Internal Medicine, State University of New York (SUNY) Downstate Health Sciences University Hospital, Brooklyn, USA; 2 Cardiology, Kings County Hospital Center, Brooklyn, USA; 3 Cardiology, Lenox Hill Hospital, Manhattan, USA

**Keywords:** stroke, cerebrovascular accident, myocardial infarction, coronary artery disease, cardiovascular disease, gastric banding, sleeve gastrectomy, roux-en-y gastric bypass, bariatric surgery, obesity

## Abstract

Obesity is a major public health problem that is associated with serious comorbidities and premature mortality. Cardiovascular disease (CVD) is the major cause of morbidity and mortality associated with obesity. Lifestyle modifications, pharmacological therapy, and weight reduction surgery are the major interventions to date available for obesity management. Bariatric surgery has been increasingly utilized as a therapeutic option for obesity. In this meta-analysis, we aim to assess the effects of bariatric surgery on CVD outcomes and cardiovascular mortality. This study was conducted in accordance with the Preferred Reporting Items for Systematic Reviews and Meta-Analyses (PRISMA) checklist. PubMed, Embase, Cochrane Library, Google Scholar, and Web of Science were searched until 03/01/2022. Our search included three types of bariatric surgery: Roux-en-Y gastric bypass (RYGB), sleeve gastrectomy, and gastric banding (GB). All were searched in conjunction with “coronary artery disease,” “ischemic heart disease,” “myocardial infarction,” “cerebrovascular accident,” “stroke,” “atrial fibrillation,” “heart failure,” “arrhythmias,” and “mortality.”

We included 49 studies meeting the study criteria. Bariatric surgery showed a beneficial effect on coronary artery disease (CAD) (hazard ratio (HR) of 0.68 {95% confidence interval (CI): 0.52-0.91}, p = 0.008), myocardial infarction (MI) (HR of 0.53 {95% CI: 0.44-0.64}, p < 0.01) heart failure (HF) (HR of 0.45 {95% CI: 0.37-0.55}, p < 0.01), cerebrovascular accident (CVA) (HR of 0.68 {95% CI: 0.59-0.78}, p < 0.01), and cardiovascular mortality (HR of 0.48 {95% CI: 0.40-0.57}, p < 0.01). The effect on atrial fibrillation (AF) did not reach statistical significance: HR of 0.81 (95% CI: 0.65-1.01), p = 0.07. Our study, that is, an updated meta-analysis, including the three types of procedure, confirms beneficial effects on the major CVD outcomes, including coronary artery disease, myocardial infarction, cerebrovascular accident, and heart failure, and on CVD mortality. This study provides updated insights into the long-term CV effects of bariatric surgery, an increasingly common intervention for obesity.

## Introduction and background

Obesity is a multifactorial disorder associated with serious complications including diabetes, dyslipidemia, cancer, and cardiovascular disease (CVD) [1,2]. Its prevalence has been uptrending over the last few decades, and it has become a modern-day epidemic [3]. Per the 2013 American Heart Association (AHA)/American College of Cardiology (ACC) guidelines, overweight is defined as a body mass index (BMI) of 25 to <30 kg/m^2^ and obesity as a BMI of 30 kg/m^2^ [4]. According to the 2017-2018 National Health and Nutrition Examination Survey (NHANES), at least two in five adults (42.4% prevalence) have obesity. This is an increase from the 1999-2000 data with a much lower prevalence of 30.5% [3]. The etiologies leading to obesity could be biological, psychosocial, socioeconomic, and environmental factors [2]. Although unhealthy dietary habits play a major role, racial differences [5] and socioeconomic factors play a major role in the high prevalence of obesity and its complications among minority populations [6]. A higher BMI was strongly associated with higher comorbid cardiovascular risk factors [1]. Of the BMI-related deaths, 41% were notably due to cardiovascular diseases [7].

Obesity is a major contributor to cardiovascular risk factors including hypertension, hyperlipidemia, coronary artery disease (CAD), heart failure (HF), stroke, sleep apnea, and arrhythmias [8]. Its pathogenesis is linked to proinflammatory factors and vessel wall remodeling, among others. Obesity accelerates atherosclerosis by promoting lipid deposition and atherothrombosis formation. It further activates the cytokines and interleukins causing endothelial dysfunction and vascular remodeling [2]. This translates into cardiovascular disease (CVD) events including CAD, myocardial infarction (MI), and stroke. Excess visceral adiposity leads to the activation of renin-angiotensin-aldosterone system, cytokine gene expression, and increased systemic circulation of proatherogenic factors [2,9]. This in turn leads to myocardial fat accumulation, increased stroke volume, cardiac wall remodeling, and fibrosis manifesting as heart failure [2,10]. Similar mechanisms lead to left atrial enlargement and fibrosis contributing to arrhythmogenesis [11].

Lifestyle modifications and increased physical activity are the initial modalities recommended in the management of obesity. Patients with a BMI of at least 40 or >35 kg/m^2^ with serious obesity-related comorbidities are considered eligible for bariatric surgery [12]. The commonly performed bariatric surgeries include sleeve gastrectomy, Roux-en-Y gastric bypass (RYGB), and gastric banding (GB) [12]. Sleeve gastrectomy is currently the most commonly performed owing to lower risk of complications. The benefits of bariatric surgery include greater long-term weight loss, reduction of major adverse cardiovascular events (MACE) [13], and cardiovascular mortality [14].

In this study, we aimed to perform an updated systematic review and a meta-analysis on bariatric surgery and major cardiovascular outcomes. The bariatric surgeries examined in our study include RYGB, sleeve gastrectomy, and gastric banding.

## Review

Methods

Literature Search and Search Strategy

This study was conducted in accordance with the Preferred Reporting Items for Systematic Reviews and Meta-Analyses (PRISMA) checklist [15]. Articles were searched online by two investigators independently through five databases and additional online sources. PubMed, Embase, Cochrane Library, Google Scholar, and Web of Science were searched at the University Hospital of Brooklyn library. Articles were restricted to only English language and searched until 03/01/2022. The search included three common types of bariatric surgery: Roux-en-Y gastric bypass, sleeve gastrectomy, and gastric banding. The search strategies included “Bariatric surgery” AND “Cardiovascular diseases,” “Roux en Y Gastric bypass” AND “Cardiovascular diseases,” “Sleeve gastrectomy” AND “Cardiovascular diseases,” and “Gastric banding” AND “Cardiovascular diseases.” Further, all three procedures were searched in conjunction with “coronary artery disease,” “ischemic heart disease,” “myocardial infarction,” “cerebrovascular accident,” “stroke,” “atrial fibrillation,” “heart failure,” and “arrhythmias.” We also reviewed prior meta-analysis articles to account for missing articles. The initial search included 3981 articles from all databases. After the removal of duplicates, 2515 articles were reviewed. A repeat search was done during manuscript writing, and additionally, one article was included in the analysis.

Study Selection and Quality Assessment

Articles were reviewed by assessing article titles and abstracts independently by two investigators (HC and TG). The intervention group included patients undergoing bariatric surgery (Roux-en-Y gastric bypass, sleeve gastrectomy, and gastric banding). The control group included non-surgical obese patients.

Patients of age >18 years and BMI of >30 kg/m^2^ with a follow-up of at least 12 months were included. Further, these studies had to include a control group and should assess at least one of the outcomes. Exclusion criteria included the following: (i) patients with malignancy, (ii) case series and conference abstracts, and (iii) studies involving cardiovascular disease cohort at baseline. But studies noting incidental cardiovascular diseases among baseline comorbid characteristics were not excluded. The quality of the studies was evaluated by the Newcastle-Ottawa Scale (NOS). Studies with less than five points carry a high risk of bias, and those with more than seven points were deemed of good quality.

Outcomes Studied

Six outcomes were studied in total. This includes CAD, MI, HF, atrial fibrillation, cerebrovascular accident (CVA), and cardiovascular disease-specific mortality. Studies assessing all-cause mortality only were excluded.

Data Extraction

**Figure 1:**
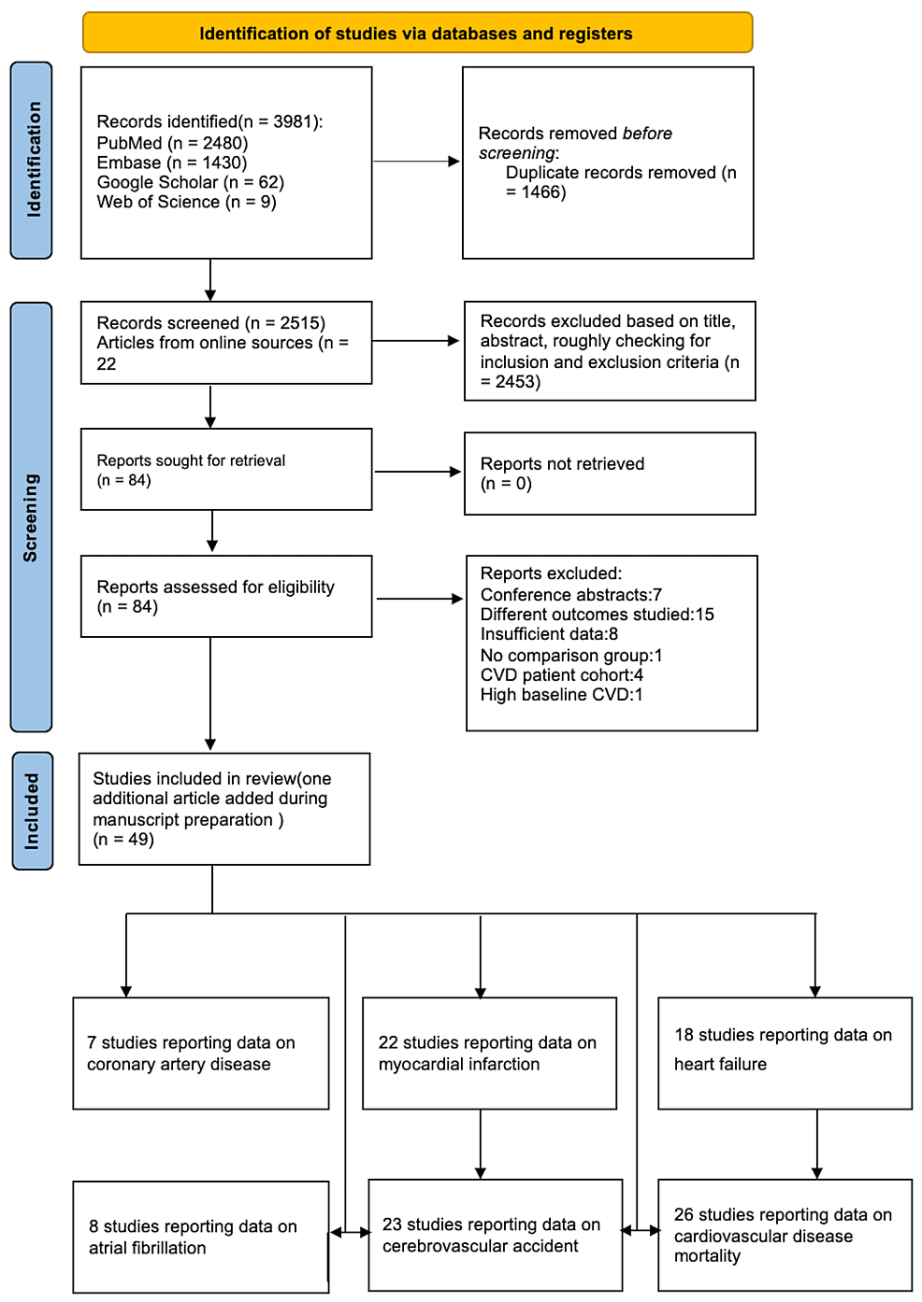
The Preferred Reporting Items for Systematic Reviews and Meta-Analyses flowchart is shown elaborating the literature databases and the study selection CVD: cardiovascular disease

Eighty-five articles were reviewed in detail, of which 49 studies were included. The reasoning for study exclusion is elaborated in the Preferred Reporting Items for Systematic Reviews and Meta-Analyses (PRISMA) flowchart (Figure 1). We extracted the following study details: sample size, gender, BMI, duration of follow-up, and end point data. The event data for intervention and control groups were obtained. Further, the adjusted and unadjusted hazard ratios (HR) with confidence intervals (CI) were extracted for the outcomes studied.

Statistical Analysis

The meta-analysis was performed with Cochrane’s Review Manager (RevMan) version 5.4. Adjusted hazard ratios were considered for the final analysis as the event rates were available for fewer studies. Hazard ratios (HR) were log transformed, and the confidence interval (CI) was used to measure standard error (SE). Genetic inverse variance and random effects model were used to obtain pooled HR and hence study the association between bariatric surgery and cardiovascular outcomes. Heterogeneity was assessed by Cochran’s Q statistic and quantified by I^2^ index. I^2^ values of <50%, 50%-75%, and >75% were considered to have low, moderate, and high heterogeneity, respectively. Publication bias was assessed using funnel plot analysis. A funnel plot was obtained for outcomes involving >10 studies.

Results

Out of the 3982 articles, 49 studies were included for data abstraction. All the included studies were cohort studies, both prospective and retrospective. Some of the studies excluded are the following: (i) studies involving malabsorptive surgery such as biliopancreatic diversion, (ii) studies that looked at outcomes in cohorts having preexisting MI and atrial fibrillation (since this would corroborate our outcome data, they were excluded), (iii) studies that had a high comorbid CVD at baseline, and (iv) studies assessing only all-cause mortality. The event rates and the hazard ratios for all the included studies are shown in Table 1.

**Table 1:** Included studies with the event rates and corresponding hazard ratios NA, not available; CI, confidence interval; HR, hazard ratio; SG, sleeve gastrectomy; RYGB, Roux-en-Y gastric bypass

Study name	Intervention group event rates	Control group event rates	Adjusted HR (CI)	Unadjusted HR (CI)
Coronary artery disease				
Bouchard et al., 2022 [13]	NA	NA	NA	NA
Fisher et al., 2018 [16]	NA	NA	0.64 (0.42-0.99)	NA
Alkharaiji et al., 2019 [17]	18/131	259/579	0.29 (0.16-0.52)	0.31 (0.19-0.52)
Aminian et al., 2019 [18]	NA	NA	0.69 (0.54-0.87)	NA
Singh et al., 2020 [19]	NA	NA	0.85 (0.61-1.19)	NA
Ardissino et al., 2021 [20]	15/593	17/593	0.6884 (0.3244-1.4610)	NA
Rassen et al., 2021 [21]	NA	NA	1.10 (0.67-1.80)	NA
Myocardial infarction				
Bouchard et al., 2022 [13]	NA	NA	NA	NA
Alkharaiji et al., 2019 [17]	13/131	95/579	0.98 (0.54-1.77)	1.03 (0.57-1.86)
Ardissino et al., 2021 [20]	6/593	6/593	NA	NA
Sampalis et al., 2006 [22]	35/1035	274/5746	0.71 (0.50-1.002)	NA
Sjöström et al., 2007 [23]	13/2010	25/2037	NA	NA
Romeo et al., 2012 [24]	NA	NA	0.56 (0.34-0.93)	NA
Sjöström et al., 2012 [25]	122/2010	136/2037	NA	0.71 (0.54-0.94)
Johnson et al., 2013 [26]	8/2580	241/13371	NA	NA
Douglas et al., 2015 [27]	5/3618	18/3732	0.28 (0.10-0.74)	NA
Eliasson et al., 2015 [28]	15/5694	39/5467	0.49 (0.24-1.01)	NA
Benotti et al., 2017 [29]	12/1724	17/1724	0.89 (0.41-1.92)	0.85 (0.41-1.79)
Brown et al., 2020 [30]	NA	NA	0.39 (0.35-0.42)	NA
Michaels et al., 2020 [31]	57/3242	323/3242	NA	NA
Moussa et al., 2020 [32]	37/3701	93/3701	0.41 (0.28-0.606)	NA
Stenberg et al., 2020 [33]	NA	NA	0.53 (0.42-0.67)	0.61 (0.50-0.75)
Wong et al., 2021 [34]	NA	NA	0.534 (0.125-2.278)	NA
Höskuldsdóttir et al., 2020 [35]	NA	NA	0.57 (0.24-1.35)	NA
Dash et al., 2021 [36]	NA	NA	0.519 (0.301-0.894)	NA
Hung et al., 2021 [37]	3/1436	15/1436	0.186 (0.054-0.643)	NA
Lundberg et al., 2021 [38]	97/28204	518/40827	0.60 (0.41-0.88)	NA
Yuan et al., 2021 [39]	NA	NA	0.24 (0.07-0.77)	0.21 (0.07-0.69)
Mentias et al., 2022 [40]	NA	NA	0.63 (0.59-0.68)	NA
Heart failure				
Bouchard et al., 2022 [13]	182/3627	377/5420	0.80 (0.70-0.90)	NA
Alkharaiji et al., 2019 [17]	13/131	91/579	0.89 (0.47-1.70)	0.81 (0.44-1.49)
Aminian et al., 2019 [18]	NA	NA	0.38 (0.30-0.49)	NA
Singh et al., 2020 [19]	NA	NA	0.57 (0.34-0.96)	NA
Rassen et al., 2021 [21]	NA	NA	0.82 (0.44-1.52)	NA
Sjöström et al., 2007 [23]	2/2010	5/2037	NA	NA
Johnson et al., 2013 [26]	35/2580	1338/13371	NA	NA
Benotti et al., 2017 [29]	24/1724	55/1724	0.38 (0.22-0.64)	0.53 (0.33-0.85)
Moussa et al., 2020 [32]	22/3701	46/3701	0.403 (0.181-0.89)	NA
Wong et al., 2021 [34]	NA	NA	0.283 (0.068-1.173)	NA
Höskuldsdóttir et al., 2020 [35]	NA	NA	0.32 (0.15-0.67)	NA
Dash et al., 2021 [36]	NA	NA	0.198 (0.109-0.36)	NA
Mentias et al., 2022 [40]	NA	NA	0.46 (0.44-0.49)	NA
Persson et al., 2017 [41]	89/22295	944/25564	0.37 (0.29-0.46)	NA
Sundström et al., 2017 [42]	44/25804	29/13701	NA	NA
Jamaly et al., 2019 [43]	188/2003	266/2030	0.66 (0.51-0.81)	0.65 (0.54-0.79)
Liakopoulos et al., 2020 [44]	86/5321	233/5321	0.33 (0.24-0.46)	NA
Höskuldsdóttir et al., 2021 [45]	47/5321	151/5321	0.27 (0.19-0.38)	NA
Atrial fibrillation				
Aminian et al., 2019 [18]	NA	NA	0.78 (0.62-0.97)	NA
Singh et al., 2020 [19]	NA	NA	0.93 (0.68-1.27)	0.94 (0.60-1.28)
Rassen et al., 2021 [21]	NA	NA	1.91 (1.10-3.33)	NA
Höskuldsdóttir et al., 2020 [35]	NA	NA	0.69 (0.30-1.62)	NA
Yuan et al., 2021 [39]	NA	NA	0.91 (0.43-1.90)	0.64 (0.31-1.31)
Höskuldsdóttir et al., 2021 [45]	104/5321	138/5321	0.59 (0.44-0.78)	NA
Jamaly et al., 2016 [46]	247/2000	340/2021	0.69 (0.58-0.82)	NA
Lynch et al., 2019 [47]	21/2522	73/2522	NA	NA
Cerebrovascular accident				
Bouchard et al., 2022 [13]	163/3627	233/5420	1.05 (0.74-1.12)	NA
Fisher et al., 2018 [16]	NA	NA	0.69 (0.38-1.25)	NA
Alkharaiji et al., 2019 [17]	8/131	40/579	0.87 (0.36-2.10)	0.77 (0.34-1.72)
Aminian et al., 2019 [18]	NA	NA	0.67 (0.48-0.94)	NA
Singh et al., 2020 [19]	NA	NA	0.98 (0.66-1.45)	NA
Ardissino et al., 2021 [20]	1/593	4/593	0.0227 (0.0009-5.45)	NA
Sjöström et al., 2007 [23]	6/2010	6/2037	NA	NA
Romeo et al., 2012 [24]	NA	NA	0.73 (0.41-1.30)	NA
Sjöström et al., 2012 [25]	93/2010	111/2037	0.66 (0.49-0.90)	NA
Johnson et al., 2013 [26]	11/2580	214/13371	NA	NA
Douglas et al., 2015 [27]	17/3683	19/3748	0.91 (0.47-1.76)	NA
Benotti et al., 2017 [29]	31/1724	49/1724	0.73 (0.45-1.17)	0.77 (0.49-1.21)
Brown et al., 2020 [30]	NA	NA	0.55 (0.51-0.59)	NA
Moussa et al., 2020 [32]	4/3701	9/3701	0.536 (0.164-1.748)	NA
Stenberg et al., 2020 [33]	NA	NA	0.81 (0.66-1.01)	0.90 (0.75-1.09)
Wong et al., 2021 [34]	NA	NA	0.811 (0.367-1.793)	NA
Höskuldsdóttir et al., 2020 [35]	NA	NA	0.18 (0.04-0.82)	NA
Dash et al., 2021 [36]	NA	NA	0.405 (0.169-0.971)	NA
Hung et al., 2021 [37]	7/1436	42/1436	0.162 (0.073-0.360)	NA
Lundberg et al., 2021 [38]	134/28204	486/40827	0.68 (0.48-0.96)	NA
Yuan et al., 2021 [39]	NA	NA	1.23 (0.64-2.35)	1 (0.53-1.91)
Mentias et al., 2022 [40]	NA	NA	0.71 (0.65-0.79)	NA
Moussa et al., 2021 [48]	19/4212	54/4212	0.352 (0.195-0.637)	NA
Cardiovascular mortality				
Carlsson et al., 2020 [14]	167/2007	221/2040	0.70 (0.57-0.85)	NA
Sjöström et al., 2007 [23]	20/2010	14/2037	NA	NA
Sjöström et al., 2012 [25]	28/2010	49/2037	0.47 (0.29-0.76)	0.56 (0.35-0.88)
Johnson et al., 2013 [26]	41/2580	985/13371	NA	NA
Eliasson et al., 2015 [28]	8/5694	33/5467	0.40 (0.15-1.05)	NA
Stenberg et al., 2020 [33]	NA	NA	NA	NA
Höskuldsdóttir et al., 2020 [35]	NA	NA	0.15 (0.03-0.68)	NA
Hung et al., 2021 [37]	0/1436	2/1436	NA	NA
Lundberg et al., 2021 [38]	196/28204	989/40827	0.78 (0.60-1.01)	NA
Liakopoulos et al., 2020 [44]	NA	NA	0.36 (0.22-0.58)	NA
Höskuldsdóttir et al., 2021 [45]	5/5321	31/5321	NA	NA
MacDonald Jr et al., 1997 [49]	2/154	12/78	NA	NA
Christou et al., 2004 [50]	49/1035	1530/5746	NA	NA
Batsis et al., 2007 [51]	4.5/173	6.8/139	NA	NA
Adams et al., 2007 [52]	55/7925	104/7925	0.51 (0.36-0.73)	0.51 (0.36-0.73)
Pontiroli et al., 2016 [53]	5/385	22/681	NA	NA
Davidson et al., 2016 [54]	NA	NA	0.51 (0.36-0.73)	NA
Lent et al., 2017 [55]	NA	NA	NA	NA
Pontiroli et al., 2018 [56]	8/154	32/360	NA	NA
Kauppila et al., 2019 [57]	525/49977	30740/494842	0.57 (0.52-0.63)	NA
Doumouras et al., 2020 [58] (RYGB)	NA	NA	0.58 (0.35-0.96)	NA
Doumouras et al., 2020 [58] (SG)	NA	NA	0.39 (0.14-1.07)	NA
Sheetz et al., 2020 [59]	NA	NA	0.47 (0.37-0.60)	NA
Courcoulas et al., 2021 [60] (RYGB)	NA	NA	0.27 (0.20-0.37)	NA
Courcoulas et al., 2021 [60] (SG)	NA	NA	0.57 (0.19-1.71)	NA
Doumouras et al., 2021 [61]	9/3041	38/3041	0.32 (0.15-0.66)	NA

Baseline study characteristics are shown in Table 2. The studies reported a mean age ranging from 32 to 62 and a mean BMI ranging from 37 to 50. All the studies were nonrandomized. Thirty-two of the studies were retrospective cohort studies, and the rest were either prospective or population-based studies.

**Table 2:** Baseline study characteristics of all included studies BMI, body mass index; CAD, coronary artery disease; ACS, acute coronary syndrome; HTN, hypertension; DM, diabetes mellitus; HbA1c, hemoglobin A1c; ESKD, end-stage kidney disease; CKD, chronic kidney disease; ASCVD, atherosclerotic cardiovascular disease; OSA, obstructive sleep apnea; MI, myocardial infarction; TIA, transient ischemic attack; IHD, ischemic heart disease; CHF, congestive heart failure; AF, atrial fibrillation; CV, cardiovascular; PAD, peripheral arterial disease; MSK, musculoskeletal; MACE, major adverse cardiovascular events; PCI, percutaneous coronary intervention; CABG, coronary artery bypass graft; LAGB, laparoscopic adjustable gastric banding; CHD, coronary heart disease; ER, emergency room; ESRD, end-stage renal disease; HD, hemodialysis; HLD, hyperlipidemia; SCORS UB-04, South Carolina Office of Research and Statistics Uniform Billing-04; ICD9, International Classification of Diseases-9; Gen, general surgery; NA, not available

Serial number	Study name	Design	Country	Type of intervention done	Study population	Inclusion criteria	Exclusion criteria	Sample size		Age (mean + SD)		BMI (mean + SD)		Follow-up duration	Primary outcome studied	Secondary outcome studied
								Intervention	Control (con)	Intervention	Control	Intervention	Control			
1	Bouchard et al., 2022 [13]	Population-based observational cohort study	Canada	Adjustable gastric banding (AGB: 42%), sleeve gastrectomy (SG: 23%), Roux-en-Y gastric bypass (RYGB: 11%), and duodenal switch (DS: 24%)	Two healthcare databases: 1) the Régie de l’Assurance Maladie du Québec (RAMQ) and 2) the Ministry of Health’s Maintenance et Exploitation des Données pour l’Étude de la Clientèle Hospitalière (MED-ÉCHO), 2007-2012	BMI of ≥35 with a comorbidity or BMI of ≥40, age of ≥18, and diagnosis of DM and/or HTN prior to the index date	Not specified	3627	5420	48 ± 10	50 ± 10	NA	NA	7.05 years	Incident composite MACE (any coronary artery event, cerebrovascular event, heart failure (HF), or all-cause mortality)	Four individual components of the primary end point
2	Carlsson et al., 2020 [14]	Prospective matched cohort study	Sweden	Vertical banded gastroplasty (69%), AGB (18%), and RYGB (13%)	The Swedish Obese Subjects (SOS), 1987-2001	Age of 37-60 years and BMI for males of ≥34 and females of ≥38	Earlier gastric/duodenal surgery (surg), ongoing malignancy, MI of <6 months, and drug/alcohol	2007	2040	47.2 ± 5.9	48.7 ± 6.3	42.4 ± 4.5	40.1 ± 4.7	Surg: 24 years; con: 22 years	All-cause mortality	CV mortality
3	Fisher et al., 2018 [16]	Retrospective cohort	USA	RYGB (76%), SG (17%), and AGB (7%)	US health plan and care delivery systems, 2005-2011	Age of 19-79 years, BMI of >35, and DM2	<1 year of enrollment, cancer, pregnancy, gestational diabetes, CAD or cerebrovascular disease, and missing BMI	5301	14934	49.5 ± 10	50.2 ± 10.1	44.7 ± 6.9	43.8 ± 6.7	Surg: 4.7 years; con: 4.6 years	Macrovascular disease	CAD and stroke separately
4	Alkharaiji et al., 2019 [17]	Retrospective cohort	UK	RYGB or SG	The Health Improvement Network (THIN), 2017	Age of >18 years and insulin-treated DM2	DM1 or non-insulin-treated DM2	131	579	50.74 ± 11.0	51.96 ± 12.8	42.77 ± 9.6	40.6 ± 9.0	10 years	Patients’ (pt) survivability against nonfatal CV events: AMI, stroke, CHD, HF, and PAD	Health covariates such as body weight, calculated BMI, HbA1c, total cholesterol, systolic/diastolic blood pressure, and likelihood of insulin independency
5	Aminian et al., 2019 [18]	Retrospective cohort	USA	RYGB (63%), SG (32%), AGB (5%), and duodenal switch (0.002%)	Cleveland Clinical Health System, 2018	Age of 18-80, BMI of ≥30, HbA1c of ≥6.5%, or ≥1 diabetic drug	Solid organ transplant, severe HF, active cancer, gastric cancer of <1 year, ER admission of <5 months, earlier gastric cancer surgery	2287	39267	52.5	61.6	45.1	35.9	3.9 years	Six-point MACE	All-cause mortality, MI, CAD, HF, stroke, AF, and neuropathy
6	Singh et al., 2020 [19]	Retrospective cohort	UK	AGB, SG, RYGB, or duodenal switch (% NA)	The Health Improvement Network (THIN), 1990-2018	>1 year registered in general practice	BMI of <30, age of >75 years, gastric cancer, gastric balloon, endo-barrier, or revisional bariatric surgery (BS)	5170	9995	45.2 ± 10.6	45.3 ± 10.5	NA	NA	3.9 years	Cardiovascular disease (CVD) (IHD, HF, stroke, and TIA), all-cause mortality, incident hypertension, and AF	All-cause mortality, IHD, HF, stroke, TIA, and AF
7	Ardissino et al., 2021 [20]	Retrospective cohort	UK	Not specified	The Clinical Practice Research Datalink (CPRD)	Age of >18 years, BMI of ≥30, and DM2	CKD of ≥3 and missing data: age, sex, BMI, and DM2	593	593	49.63	49.47	45.54	45.14	42.7 months	ASCVD	All-cause mortality, CAD, stroke, and PAD
8	Rassen et al., 2021 [21]	Retrospective cohort	USA	RYGB (50%), SG (44%), and gastric resection (8%)	Electronic health records licenced from Optum, 2007-2018	Age of 18-80 years, DM2, and BMI of ≥30	Solid organ transplant, severe HF, cancer in the past year, peptic ulcer disease on the index date, and ER admission of five prior to index date	344	551	57.9	59	42.6	42.1	Surg: 2.7 years; con: 2.4 years	Six-point MACE, B12 deficiency, anemia, and cholelithiasis	Not specified
9	Sampalis et al., 2006 [22]	Retrospective cohort	Canada	RYGB (81.3%) and vertical banded gastroplasty (18.7%)	McGill University Health Centre, 1986-2002	Not specified other than BS	Cancer, hematological disease, CVD, digestive diseases, endocrinologic disease including diabetes and genitourinary, infectious, musculoskeletal, nervous system, psychiatric and mental, respiratory, and skin diseases	1035	5746	45 ± 12	47 ± 13	NA	NA	2.5 years	Incidence of CV- and MSK-related conditions and treatments	Not specified
10	Sjöström et al., 2007 [23]	Prospective matched cohort	Sweden	Vertical banded gastroplasty (68%), AGB (19%), and RYGB (13%)	The Swedish Obese Subjects, 1987-2001	Age of 37-60 years, BMI for males of ≥34 and females of ≥38	Not specified	2010	2037	46.1 ± 5.8	47.4 ± 6.1	41.8 ± 4.4	40.9 ± 4.3	10.9 ± 3.5	All-cause mortality	Not specified
11	Romeo et al., 2012 [24]	Prospective, nonrandomized, controlled interventional trial	Sweden	RYGB (16%), gastric banding (18%), and vertical gastroplasty (66%)	The Swedish Obese Subjects (SOS), 1987-2001	DM2, age of 37- 60, and BMI of ≥34 for males and ≥38 for females	Earlier gastric/duodenal ulcer surgery; earlier bariatric surgery; gastric ulcer/MI in the past six months; ongoing/active malignancy in the past five years; bulimic, drug/alcohol, psychiatric, or cooperative problems contraindicating bariatric surgery; and other contraindicating conditions, such as continuous glucocorticoid or anti-inflammatory treatment	345	262	49 ± 6	50 ± 6	42 ± 5	40 ± 5	13.3 years	CV events (MI and stroke, whichever came first), as well as MI and stroke analyzed separately	Not specified
12	Sjöström et al., 2012 [25]	Prospective matched cohort	Sweden	Gastric bypass (13.2%), banding (18.7%), or vertical banded gastroplasty (68.1%)	The Swedish Obese Subjects (SOS), 1987-2001	Age of 37-60 years and BMI for males of ≥34 and females of ≥38	Earlier gastric/duodenal ulcer surgery; earlier bariatric surgery; gastric ulcer/MI in the past six months; ongoing/active malignancy in the past five years; bulimic, drug/alcohol, psychiatric, or cooperative problems contraindicating bariatric surgery; and other contraindicating conditions, such as continuous glucocorticoid or anti-inflammatory treatment	2010	2037	46.1	47.8	42.4	40.1	14.7 years	Total mortality	MI and stroke
13	Johnson et al., 2013 [26]	Retrospective cohort	USA	Gastric bypass, adjustable gastric banding, vertical banded gastroplasty, or biliopancreatic diversion or sleeve gastrectomy	SCORS UB-04	Moderate and severely obese patients with DM2, age of 18-77 years, and no documented history of (h/o) MI, angina, CHF, stroke, or advanced microvascular disease (previous nontraumatic amputation, laser eye/retinal surgery, blindness in at least one eye, ESRD, or creation of arteriovenous (AV) access for HD)	Type 1 diabetes, did not have diagnosis code specific to moderate or severe obesity, or had missing or incompatible data	2580	13371	47.5 ± 10.6	52.1 ± 12.8	NA	NA	Surg: 1.768 years; con: 1.58 years	Macrovascular (acute MI, stroke, or all-cause death) or microvascular (new diagnosis of blindness in at least one eye, laser eye or retinal surgery, nontraumatic amputation, or creation of permanent arteriovenous access for dialysis)	Macrovascular and microvascular complications considered separately, as well as other vascular complications, including revascularization of coronary, carotid, or lower extremity arteries or a new diagnosis of congestive heart failure or angina pectoris
14	Douglas et al., 2015 [27]	Retrospective cohort	UK	AGB (47.1%), RYGB (36.6%), SG (15.8%), and others (0.5%)	Clinical Practice Research Datalink, 2014	>12 months of prior registration in database	Skin cancer and missing BMI data/BMI <35	3882	3882	45 ± 11	45 ± 11	44.7 ± 8.8	42.1 ± 6.5	3.4 years	Weight, BMI, DM2, HTN, angina, MI, stroke, fractures, OSA and cancer, mortality, and resolution of hypertension and DM2	All-cause mortality and stroke
15	Eliasson et al., 2015 [28]	Retrospective cohort	Sweden	RYGB (100%)	National Diabetes Register and Scandinavian Obesity Surgery Registry, 2007-2014	Age between 18 and 60 years	Not specified	6132	6132	48.4 ± 9.8	50.5 ± 12.7	42 ± 5.7	41.4 ± 5.7	3.5 years	Total mortality, cardiovascular mortality, and fatal or nonfatal MI	MI and CV mortality
16	Benotti et al., 2017 [29]	Retrospective cohort	USA	RYGB (100%)	Geisinger Health Center, 2002-2012	Age of 20-80 years, BMI of >35, and no preexisting CVD (ICD9 410-449)	Missing data to calculate Framingham Risk Score	1724	1724	45.0 ± 10.6	45.1 ± 10.6	46.5 ± 6.0	46.5 ± 6.1	6.3 years	Combined MI/HF/stroke	Stroke, MI, and HF
17	Brown et al., 2020 [30]	Retrospective cohort	USA	RYGB (52.19%), SG (13.81%), and AGB (34%)	Statewide Planning and Research Cooperative System database, 2006-2012	Age of ≥18 years	In-hospital death in earliest record, age of <18 years, duplicated records, and missing or unknown gender	60445	268362	42.72 ± 11.55	43.28 ± 11.75	NA	NA	Not specified	Any type of CV event, MI, and stroke	Cardiovascular events
18	Michaels et al., 2020 [31]	Retrospective cohort	USA	RYGB (78.9%), AGB (11.7%), SG (7.7%), and others (1.7%)	Single Virginia Academic Hospital, 1985-2015	Not specified other than BS	Not specified	3242	3242	43	43	47.7	48	Surg: 6.1 years; con: 8.1 years	Incident MI, coronary catheterization, PCI, and CABG	Not specified
19	Moussa et al., 2020 [32]	Prospective cohort	UK	RYGB (38%), AGB (35%), SG (15%), others (1%), and undefined (11%)	UK Clinical Practice Research Datalink, 2020	Not specified other than BS	BMI of <35, MACE before index date, lost to follow-up <12 months after index date, and missing data: age, BMI, and sex	3701	3701	36	36	40.5	40.3	140.7 months	Combined MI/stroke	All-cause mortality, MI, stroke, and HF
20	Stenberg et al., 2020 [33]	Retrospective matched cohort	Sweden	RYGB (90.1%) and sleeve gastrectomy (9.9%)	Scandinavian Obesity Surgery Register (SOReg) and the Swedish National Patient Registers (NPR)	Not specified other than BS	<18 years, without HTN, those with antihypertensive therapy possibly for other reasons, and pt without at least one matched control with HTN	11863	26199	52.1 ± 7.46	54.6 ± 7.12	41.9 ± 5.43	NA	Surg: 61.1 ± 30.4 months; con: 60.7 ± 30.6 months	MACE	ACS, cerebrovascular event, all-cause mortality, CV mortality, and remission of HTN
21	Wong et al., 2021 [34]	Retrospective matched cohort study	China	Sleeve gastroplasty (80.5%), RYGB (16.2%), and revision procedure (3%)	Hospital Authority database in the Hong Kong adult diabetes population, 2006-2017	DM2	BMI of <27.5, non-DM2, history of CVD, and eGFR of <30	303	1399	51.35 ± 12.26	50.98 ± 13.44	37.44 ± 5.04	36.55 ± 6.49	32 months	All-cause mortality, composite CVD events (acute MI, other IHD, CHF, stroke, and PVD), ESKD, and severe hypoglycemia	NA
22	Höskuldsdóttir et al., 2020 [35]	Nationwide, matched, observational cohort study	Sweden	RYGB (100%)	National Diabetes Register and Scandinavian Obesity Surgery Registry, 2007-2013	DM1	Not specified	387	387	41.7 ± 10.3	41.1 ± 14.5	40.8 ± 5.4	39.5 ± 7.0	9 years	All-cause mortality, CV disease, stroke, HF, and hospitalization for serious hypo- or hyperglycemic events, amputation, psychiatric disorders, changes in kidney function, and substance abuse	Not specified
23	Dash et al., 2021 [36]	Retrospective cohort study	Canada	RYGB (92.7%) and SG (7.3%)	University Health Network (UHN), 2008-2017	BMI of ≥40 or ≥35 with comorbidities	Not Ontario residents, those who had surgery either before or >2.5 years after their referral date to the UHN bariatric program, and those who underwent procedures other than RYGB or SG, ineligible for surgery	3098	5470	43.19 ± 10.36	46.15 ± 12.13	47.92 ± 8.07	47.37 ± 11.53	NA	Stroke, MI, CHF hospitalization, or death	MI, stroke, HF, coronary revascularization, carotid revascularization, all-cause mortality, and hospitalization for chronic kidney disease, chronic liver disease, and psychiatric disease
24	Hung et al., 2021 [37]	Retrospective cohort	Taiwan	Gastric banding (13.87%), gastric bypass (52.68%), one-anastomosis gastric bypass (3.49%), and laparoscopic sleeve gastrectomy (29.87%)	Taiwan National Health Insurance Research Database (NHIRD), 2003-2008	Age of 18-55 years and BMI of >35 kg/m^2^ with comorbidities or >40 kg/m^2^	A primary diagnosis of any condition other than obesity, died during admission or within 30 days following the index admission, had a history of any CV disease, had undetermined sex, and were diagnosed with gastric malignancy	1436	1436	32.39 ± 8.63	32.27 ± 9.25	NA	NA	89.65 months	Incidence of CV events	Not specified
25	Lundberg et al., 2021 [38]	Prospective cohort	Sweden	RYGB (100%)	Swedish National Patient Registry, 2001-2013	Age of 20-65 years and BMI of ≥35	Other bariatric surgery or died <2 years after obesity diagnosis	28 204	40 827	40.8 ± 10.4	43.1 ± 11.8	NA	NA	Surg: 4 years; con: 4.8 years	All-cause mortality, MI, ischemic stroke, and cardiovascular-related mortality	Not specified
26	Yuan et al., 2021 [39]	Retrospective cohort	USA	RYGB (100%)	Obesity clinic at Mayo Clinic, Rochester, MN, 1993-2012	BMI of >35	Pts underwent gastric banding and incomplete data	308	701	44.2 ± 10.5	43.6 ± 12.6	46.4 ± 6.5	44.8 ± 6.9	1 year of index diagnosis	New-onset AF	MACE
27	Mentias et al., 2022 [40]	Prospective cohort	USA	SG (65.5%), gastric bypass (33.3%), and gastric banding (1.3%)	Medicare beneficiaries through 2013-2019	Medicare beneficiaries enrolled in part A	>75 years, history of established HF, and enrolled in Medicare for <1 year before the study entry date. Patients that had an emergent/urgent admission, are admitted to a skilled nursing facility or long-term acute care, and are discharged to any destination other than home	94885	94885	62.33 ± 10.62	62.33 ± 10.62	44.71 ± 7.3	44.71 ± 7.3	4 years	All-cause mortality	Time to admission with a diagnosis of new-onset HF, MI, and ischemic stroke. Secondary outcomes also included total rate of admissions with HF in follow-up
28	Persson et al., 2017 [41]	Retrospective cohort	Sweden	RYGB (92.8%), gastric banding (3.5%), vertical banded gastroplasty (3%), and gastroduodenal bypass (0.7%)	Swedish National Patient Registry, 2000-2011	Age of 18-74 years with first recorded diagnosis of obesity	HF at or before obesity diagnosis and died on the same time of obesity diagnosis	22295	25564	40.7 ± 10.7	44.3 ± 13.2	NA	NA	3.7 years	Incident HF and mortality	Not specified
29	Sundström et al., 2017 [42]	Prospective cohort	Sweden	RYGB (100%)	Scandinavian Obesity Surgery Registry (2007-2012) and Itrim health database (2006-2013)	BMI of 30-49.9 and age of ≥18 years	Crossover, HF at baseline, and missing data on education or marital status	25804	13701	41.3	41.5	41.5	41.4	4.1 years	Incident HF	Nonischemic HF
30	Jamaly et al., 2019 [43]	Prospective matched cohort	Sweden	Vertical banded gastroplasty (68%), AGB (19%), and RYGB (13%)	The Swedish Obese Subjects, 1987-2001	Age of 37-60 years and BMI for males of ≥34 and females of ≥38	Earlier gastric/duodenal ulcer surgery; earlier bariatric surgery; gastric ulcer/MI in the past six months; ongoing/active malignancy in the past five years; bulimic, drug/alcohol, psychiatric, or cooperative problems contraindicating bariatric surgery; and other contraindicating conditions, such as continuous glucocorticoid or anti-inflammatory treatment	2003	2030	47.2 ± 5.9	48.7 ± 6.3	42.4 ± 4.5	40.1 ± 4.7	22 years	Incident HF	Not specified
31	Liakopoulos et al., 2020 [44]	Retrospective observational cohort	Sweden	Gastric bypass	National Diabetes Register and the Scandinavian Obesity Surgery Register, 2007-2015	Age of 18-75 years and DM2	Not specified	5321	5321	49 ± 9.5	47.1 ± 11.5	42 ± 5.7	40.9 ± 7.3	Surg: 4.7 years; con: 4.6 years	Incident renal disease	CV diagnoses, heart failure, and mortality
32	Höskuldsdóttir et al., 2021 [45]	Nationwide, matched, observational cohort study	Sweden	RYGB (100%)	National Diabetes Register and Scandinavian Obesity Surgery Registry, 2007-2013	Age of 18-65 years, BMI of >27.5, and DM2	Procedures other than RYGB	5321	5321	48.96 ± 9.50	47.14 ± 11.49	42.03 ± 5.65	40.95 ± 7.30	4.5 years	Hospitalization for HF and/or AF and mortality in patients with preexisting HF	Not specified
33	Jamaly et al., 2016 [46]	Prospective matched cohort	Sweden	Vertical banded gastroplasty (68%), AGB (19%), and RYGB (13%)	The Swedish Obese Subjects, 1987-2001	Age of 37-60 years, BMI for males of ≥34 and females of ≥38	H/o AF at baseline, gastric surgery, ongoing malignancy, recent myocardial infarction, a bulimic eating pattern, alcohol/drug abuse, or psychiatric problems likely to impair study compliance	2000	2021	47.2 ± 5.9	48.6 ± 6.2	42.4 ± 4.5	40.1 ± 4.7	19 years	Incident AF	Not specified
34	Lynch et al., 2019 [47]	Retrospective cohort	USA	RYGB or SG (% NA)	Single Virginia Academic Hospital, 1985-2015	Age of >18 years	Banded gastroplasty pts and preexisting AF	2522	2522	42	42	47.1	47.7	Surg: 6.2 years; con: 8.0 years	Incident AF	Not specified
35	Moussa et al., 2021 [48]	Retrospective cohort	UK	NA	UK Clinical Practice Research Datalink, 2021	Not specified other than BS	Had primary event before enrollment	4212	4212	50	51	40.4	40.5	11.4 years	Cerebrovascular event	Ischemic events, hemorrhagic events, individual components of the primary end point alone, and all-cause mortality
36	MacDonald Jr et al., 1997 [49]	Retrospective cohort	USA	RYGB (100%)	Obesity Research Program at East Carolina University, 1979-1994	Non-insulin-dependent DM2	No non-insulin-dependent DM2, no morbid obesity, and age of >64 years	154	78	41.9	43.5	50.6	48.8	Surg: 9 years; con: 6.2 years	All-cause mortality	Not specified
37	Christou et al., 2004 [50]	Observational two-cohort study	Canada	RYGB (79.2%), vertical banded gastroplasty (18.7%), and laparoscopic RY isolated gastric bypass (2.2%)	McGill University Health Centre between 1986 and 2002	Not specified other than BS	Subjects with medical conditions (other than morbid obesity) at cohort inception into the study	1035	5746	45.1 ± 11.6	46.7 ± 13.1	NA	NA	Surg: 2.5 years; con: 2.6 years	Long-term mortality, morbidity, and healthcare use	Not specified
38	Batsis et al., 2007 [51]	Population-based, historical cohort	USA	RYGB (100%)	Mayo Clinic medical record, the Mayo Surgical Index, and the Rochester Epidemiology Project (REP), 1990-2003	Not specified other than RYGB	Missing data and BMI of <35	197	163	44.0 ± 9.9	43.4 ± 11.2	49.5 ± 8.9	44 ± 5.7	3.3 years	All-cause mortality, cardiovascular mortality, cardiovascular events, and combined cardiovascular events/all-cause mortality	Not specified
39	Adams et al., 2007 [52]	Retrospective cohort	USA	RYGB (100%)	Single Utah surgical practice, 1984-2002	Not specified	Not specified	7925	7925	39.5 ± 10.5	39.3 ± 10.6	45.3 ± 7.4	46.7 ± 6.3	7.1 years	Death from any cause	Death from various specific causes: all deaths caused by disease: CV disease (HF, CAD, stroke, and other CV), diabetes, cancer, other diseases. All non-disease causes: accident unrelated to drugs, poisoning of undetermined intent, suicide, and others
40	Davidson et al., 2016 [54]	Retrospective cohort	USA	RYGB (100%)	Private surgical practice, Utah, 1984-2002	Not specified other than BS	Not specified	7925	7925	39.5 ± 10.5	39.5 ± 10.6	45.3 ± 7.4	46.7 ± 6.3	7.2 years	All-cause and cause-specific mortality	Not specified
41	Lent et al., 2017 [55]	Retrospective observational cohort	USA	RYGB (100%)	A large comprehensive medical center, 2004-2015	Age of 18-70 years, BMI of >40 kg/m^2^ (or >35 kg/m^2^ with comorbidity of DM, HTN, HLD, or OSA), active in the primary care system for an extended period of time (three or more office visits over >2-year period), no prior h/o bariatric surgery, and no diagnosis of serious mental health disorders or illegal drug use	Surgery other than RYGB	DM: 625; no DM: 1803	DM: 625, no DM: 1803	DM: 52.5 ± 9.4; no DM: 48.3 ± 11	DM: 52.5 ± 9.4; no DM: 43.9 ± 11	DM: 44.9 ± 6.0; no DM: 47.4 ± 6.4	DM: 44.9 ± 6.1; no DM: 47.3 ± 6.4	5.8 years	All-cause mortality, stratified by “baseline” diabetes status	Cause-specific mortality, stratified by “baseline” diabetes status
42	Pontiroli et al., 2018 [56]	Retrospective cohort	Italy	LAGB (100%)	Italian National Health System Lumbardy database (LAGB10 study group), 1995-2001	BMI of ≥40 or ≥35 with comorbidities and age of 18-65 years	Not specified	154	360	41.0 ± 10.13	42.2 ± 12.94	42.7 ± 4.62	39.1 ± 5.27	19.5 ± 1.87 years	All-cause mortality	Not specified
43	Kauppila et al., 2019 [57]	Population-based cohort	Denmark, Finland, Iceland, Norway, and Sweden	Gastric bypass (73.4%), vertical banded gastroplasty (11%), gastric banding (10.9%), other restrictive procedures (3.2%), or blocking procedures (1.5%)	Nordic Obesity Surgery Cohort (NordOSCO)	Not specified other than BS	Not specified	49977	494842			NA	NA	>15 years	All-cause mortality	Mortality, specifically in the obesity-related morbidities, cardiovascular disease, diabetes, cancer, and suicide
44	Doumouras et al., 2020 [58] (RYGB)	Population-based matched cohort	Canada	RYGB (87%) and sleeve gastrectomy (13%)	The Ontario Bariatric Network (OBN), 2010-2016	Not specified other than BS	Non-Ontario residents, age of ≥70 years, BMI of 35 kg/m^2^ or less, h/o cancer within two years, active substance use disorder, accessed palliative care, pregnancy as of the index date, previous solid organ transplantation, active cardiac disease or major revascularization procedure within six months of the index date, or severe liver disease with ascites within one year of the index date	13679	13679	45.23 ± 10.89	45.49 ± 11.63	47.21 ± 8.01	46.70 ± 8.44	Gen: 4.89 years; con: 4.84 years	All-cause mortality	Cause-specific mortality
45	Doumouras et al., 2020 [58] (SG)	Population-based matched cohort	Canada	RYGB (87%) and sleeve gastrectomy (13%)	The Ontario Bariatric Network (OBN), 2010-2016	Not specified other than BS	Non-Ontario residents, age of ≥70 years, BMI of 35 kg/m^2^ or less, h/o cancer within two years, active substance use disorder, accessed palliative care, pregnancy as of the index date, previous solid organ transplantation, active cardiac disease or major revascularization procedure within six months of the index date, or severe liver disease with ascites within one year of the index date	13679	13679	45.23 ± 10.89	45.49 ± 11.63	47.21 ± 8.01	46.70 ± 8.44	Gen: 4.89 years; con: 4.84 years	All-cause mortality	Cause-specific mortality
46	Sheetz et al., 2020 [59]	Retrospective cohort	USA	Sleeve gastrectomy (45.1%), Roux-en-Y gastric bypass (41.6%), gastric banding (12.8%), or duodenal switch (0.4%)	US Renal Data System registry, 2006-2015	Not specified other than BS	<18 years, similarly coded surgery for a diagnosis of malignancy, BMI of <35, or without a recorded BMI	1597	4750	49.8 ± 11.2	51.7 ± 11.1	45.6 ± 6.7	44.6 ± 6.8	3 years	All-cause mortality at five years	Disease-specific mortality and incidence of kidney transplant
47	Courcoulas et al., 2021 [60] (RYGB)	Retrospective matched cohort	USA	SG (45%) and RYGB (55%)	Kaiser Permanente regions Washington and California, 2005-2015	Age of 19-79 years and BMI of ≥35	<1 year of enrollment, pregnancy, h/o cancer (except non-melanoma skin cancer, and missing BMI data/BMI of <35							4.9 years	All-cause mortality	CV, cancer, and diabetes-related health
48	Courcoulas et al., 2021 [60] (SG)	Retrospective matched cohort	USA	SG (45%) and RYGB (55%)	Kaiser Permanente regions Washington and California, 2005-2015	Age of 19-79 years and BMI of ≥35	<1 year of enrollment, pregnancy, and h/o cancer (except non-melanoma							4.9 years	All-cause mortality	CV, cancer, and diabetes-related health
49	Doumouras et al., 2021 [61]	Retrospective matched cohort	Canada	RYGB (86.7%) and sleeve gastrectomy (13.3%)	Ontario ICES database, 2010-2016	DM2 and BMI of ≥35	Non-Ontario pts, BMI of <35, age of ≥70 years, h/o cancer within two years, active substance abuse, had accessed palliative care, pregnant, had previous solid organ transplantation, had active cardiac disease or major revascularization procedure within six months of index date, or had severe liver disease with ascites within one year of the index date	3455	3455	51.66 ± 9.20	52.41 ± 9.67	45.29 ± 7.55	44.06 ± 8.25	4.6 years	All-cause mortality	Cause-specific mortality and nonfatal morbidities

Using the Newcastle-Ottawa Scale (NOS), studies were assessed for quality, of which all studies had at least a score of 7 and none were excluded. The quality assessment of the studies can be found in Table 3.

**Table 3:** Quality assessment of studies using the Newcastle-Ottawa Scale (NOS) AHRQ: Agency for Healthcare Research and Quality

Study name	Study type	Selection	Comparability	Exposure	Total score	AHRQ standards
Bouchard et al., 2022 [13]	Cohort	3	2	3	8	Good quality
Carlsson et al., 2020 [14]	Cohort	3	2	3	8	Good quality
Fisher et al., 2018 [16]	Cohort	4	2	3	9	Good quality
Alkharaiji et al., 2019 [17]	Cohort	4	2	2	8	Good quality
Aminian et al., 2019 [18]	Cohort	4	2	3	9	Good quality
Singh et al., 2020 [19]	Cohort	3	2	2	7	Good quality
Ardissino et al., 2021 [20]	Cohort	3	2	2	7	Good quality
Rassen et al., 2021 [21]	Cohort	4	2	3	9	Good quality
Sampalis et al., 2006 [22]	Cohort	4	2	2	8	Good quality
Sjöström et al., 2007 [23]	Cohort	4	2	3	9	Good quality
Romeo et al., 2012 [24]	Cohort	4	2	2	8	Good quality
Sjöström et al., 2012 [25]	Cohort	4	2	3	9	Good quality
Johnson et al., 2013 [26]	Cohort	4	2	3	9	Good quality
Douglas et al., 2015 [27]	Cohort	4	2	3	9	Good quality
Eliasson et al., 2015 [28]	Cohort	4	2	3	9	Good quality
Benotti et al., 2017 [29]	Cohort	4	2	3	9	Good quality
Brown et al., 2020 [30]	Cohort	4	2	2	8	Good quality
Michaels et al., 2020 [31]	Cohort	3	2	2	7	Good quality
Moussa et al., 2020 [32]	Cohort	3	2	2	7	Good quality
Stenberg et al., 2020 [33]	Cohort	4	2	3	9	Good quality
Wong et al., 2021 [34]	Cohort	3	2	2	7	Good quality
Höskuldsdóttir et al., 2020 [35]	Cohort	3	2	2	7	Good quality
Dash et al., 2021 [36]	Cohort	2	2	3	7	Good quality
Hung et al., 2021 [37]	Cohort	4	2	2	8	Good quality
Lundberg et al., 2021 [38]	Cohort	4	2	2	8	Good quality
Yuan et al., 2021 [39]	Cohort	4	2	2	8	Good quality
Mentias et al., 2022 [40]	Cohort	3	2	3	8	Good quality
Persson et al., 2017 [41]	Cohort	4	2	2	8	Good quality
Sundström et al., 2017 [42]	Cohort	4	2	2	8	Good quality
Jamaly et al., 2019 [43]	Cohort	4	2	2	8	Good quality
Liakopoulos et al., 2020 [44]	Cohort	3	2	2	7	Good quality
Höskuldsdóttir et al., 2021 [45]	Cohort	4	2	3	9	Good quality
Jamaly et al., 2016 [46]	Cohort	4	2	3	9	Good quality
Lynch et al., 2019 [47]	Cohort	4	2	3	9	Good quality
Moussa et al., 2021 [48]	Cohort	4	2	3	9	Good quality
Macdonald Jr et al., 1997 [49]	Cohort	3	2	2	7	Good quality
Christou et al., 2004 [50]	Cohort	4	2	3	9	Good quality
Batsis et al., 2007 [51]	Cohort	3	2	3	8	Good quality
Adams et al., 2007 [52]	Cohort	3	2	3	8	Good quality
Davidson et al., 2016 [54]	Cohort	3	2	2	7	Good quality
Lent et al., 2017 [55]	Cohort	4	2	3	9	Good quality
Pontiroli et al., 2018 [56]	Cohort	4	2	2	8	Good quality
Kauppila et al., 2019 [57]	Cohort	3	2	3	8	Good quality
Doumouras et al., 2020 [58] (RYGB)	Cohort	4	2	2	8	Good quality
Doumouras et al., 2020 [58] (SG)	Cohort	4	2	2	8	Good quality
Sheetz et al., 2020 [59]	Cohort	3	2	3	8	Good quality
Courcoulas et al., 2021 [60] (RYGB)	Cohort	3	2	2	7	Good quality
Courcoulas et al., 2021 [60] (SG)	Cohort	3	2	2	7	Good quality
Doumouras et al., 2021 [61]	Cohort	4	2	3	9	Good quality

Effect on Coronary Artery Disease

**Figure 2:**
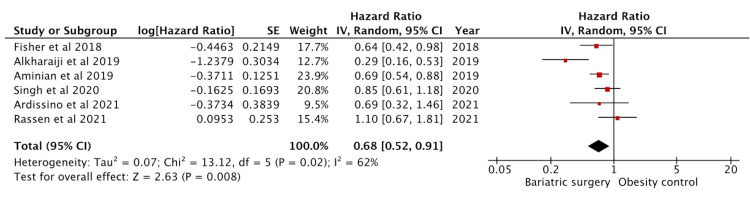
Forest plot with the included studies and the pooled hazard ratio for coronary artery disease CI: confidence interval Sources: [13,16-21]

Seven studies reported effects on CAD, of which six had adjusted HR ratio data and were included in the analysis (Figure 2). One study by Bouchard et al. [13] reported a combined HR for both CAD and MI. Since individual data were not available, it was excluded from the analysis to avoid duplication of data and bias. Of the included studies, there were 17423 bariatric surgery patients and 43507 controls. The effect on CAD was significant with a pooled HR of 0.68 (95% CI: 0.52-0.91) (p = 0.008).

Effect on Myocardial Infarction

**Figure 3:**
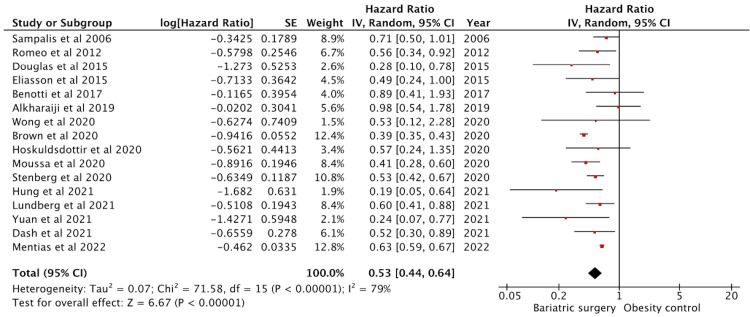
Forest plot with the included studies and the pooled hazard ratio for myocardial infarction CI: confidence interval Sources: [13,17,20,22-35,40]

Twenty-two studies reported myocardial infarction outcomes. Sixteen studies had adjusted HR data and were included in the analysis (Figure 3). These studies had 231503 patients in the intervention group and 487727 in the control group. The effect on MI was significant with a pooled HR of 0.53 (95% CI: 0.44-0.64) (p < 0.01). The studies showed high heterogeneity with an I^2^ = 79%.

Like previously mentioned, Bouchard et al. [13] reported a combined incidence and hence was excluded. Johnson et al. [26], Sjöström et al. [23,25], Michaels et al. [31], and Ardissino et al. [20] provided only the incidence data and were not included in the analysis. Naslund et al. [62] studied the outcomes in patients with preexisting MI and was excluded.

Effect on Heart Failure

**Figure 4:**
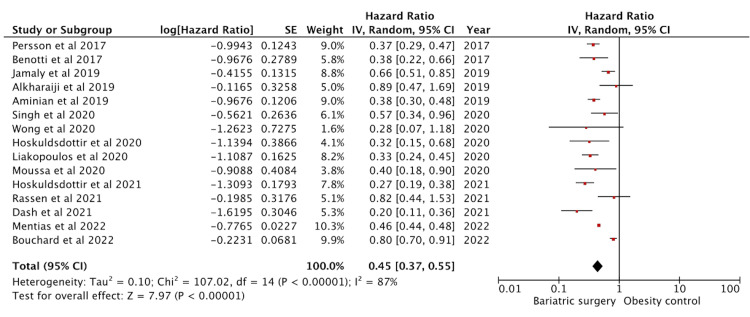
Forest plot with the included studies and the pooled hazard ratio for heart failure CI: confidence interval Sources: [13,17-19,21,23,26,29,32-36,40-45]

Eighteen studies reported heart failure outcomes. Fifteen studies had adjusted HR data and were included in the analysis (Figure 4). These studies amounted to a sample size of 180961 in the intervention group and 202891 in the control group. The effect on heart failure was significant with a pooled HR of 0.45 (95% CI: 0.37-0.55) (p < 0.01). The studies showed high heterogeneity with an I^2^ = 87%. Sundström et al. [42], Johnson et al. [26], and Sjöström et al. [23] had only provided relative risk data and were excluded from the analysis.

Effect on Atrial Fibrillation

**Figure 5:**
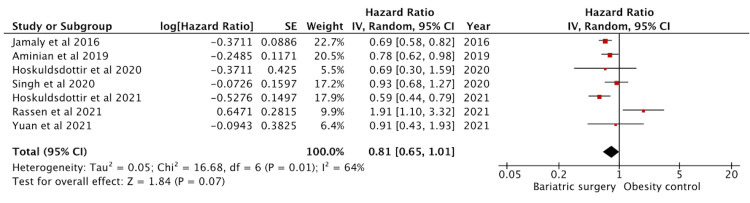
Forest plot with the included studies and the pooled hazard ratio for atrial fibrillation CI: confidence interval Sources: [18,19,21,35,39,45-47]

Eight studies reported atrial fibrillation outcomes. Seven had adjusted HR data and were included in the analysis (Figure 5). These studies amounted to a sample size of 18309 in the intervention group and 32933 in the control group. The effect on atrial fibrillation was not significant with a pooled HR of 0.81 (95% CI: 0.65-1.01) (p = 0.07). Lynch et al. [47] provided relative risk data only and hence was excluded.

Effect on Cerebrovascular Accident

**Figure 6:**
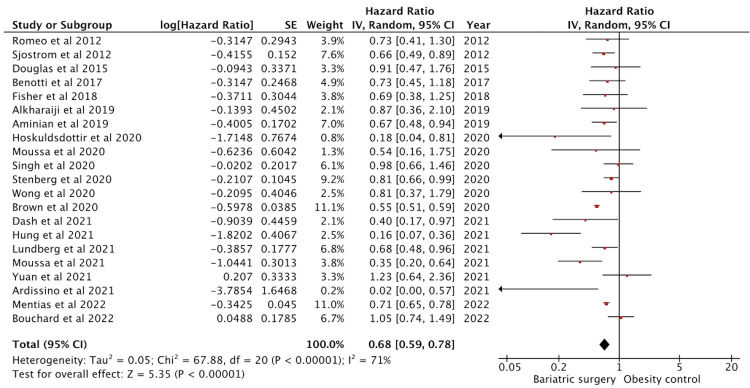
Forest plot with the included studies and the pooled hazard ratio for cerebrovascular accident CI: confidence interval Sources: [13,16-20,23-27,29,30,32-40,48]

Twenty-three studies reported cerebrovascular accident (CVA) outcomes. Twenty-one studies had adjusted HR data and were included in the analysis (Figure 6). These studies amounted to a sample size of 238472 subjects and 513848 controls. The effect on CVA was significant with a pooled HR of 0.68 (95% CI: 0.59-0.78) (p < 0.01). The studies showed moderate heterogeneity with an I^2^ = 72%. 

Johnson et al. [26] and Sjöström et al. [23] reported data for relative risks only and were excluded. Some studies for CVA reported ischemic outcomes (transient ischemic attack and ischemic stroke) and hemorrhagic outcomes (hemorrhagic stroke and intraparenchymal hemorrhage) separately. We have not made differentiation between the entities and have reported it as a composite CVA outcome.

Effect on Cardiovascular Mortality

**Figure 7:**
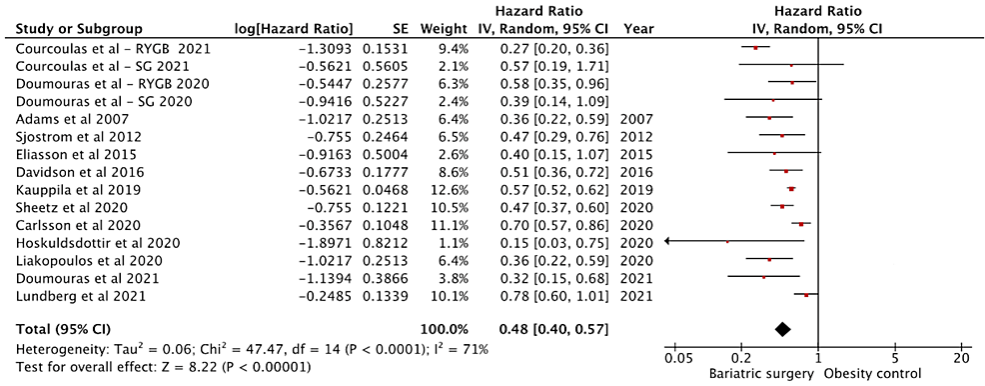
Forest plot with the included studies and the pooled hazard ratio for cardiovascular mortality CI: confidence interval Sources: [14,23,25-28,33,35-38,44,45,49-61]

Twenty-six studies reported cardiovascular disease-specific mortality. Fifteen studies had adjusted HR data and were included in the analysis (Figure 7). There were 157750 in the surgery group and 643770 in the control groups. The effect on cardiovascular disease (CVD) mortality was significant with a pooled HR of 0.48 (95% CI: 0.40-0.57) (p < 0.01). The studies showed high heterogeneity with an I^2^ = 71%.

Pontiroli et al. [53,56], Höskuldsdóttir et al. [35,45], Stenberg et al. [33], Sjöström et al. [23], MacDonald Jr et al. [49], Lent et al. [55], Hung et al. [37], Batsis et al. [51], Johnson et al. [26], and Christou et al. [50] had insufficient data for hazard ratio and were excluded. Courcoulas et al. [60] and Doumouras et al. [58] studied CVD data separately on sleeve gastrectomy and RYGB. Hence, they were included as separate outcomes.

Publication bias

**Figure 8:**
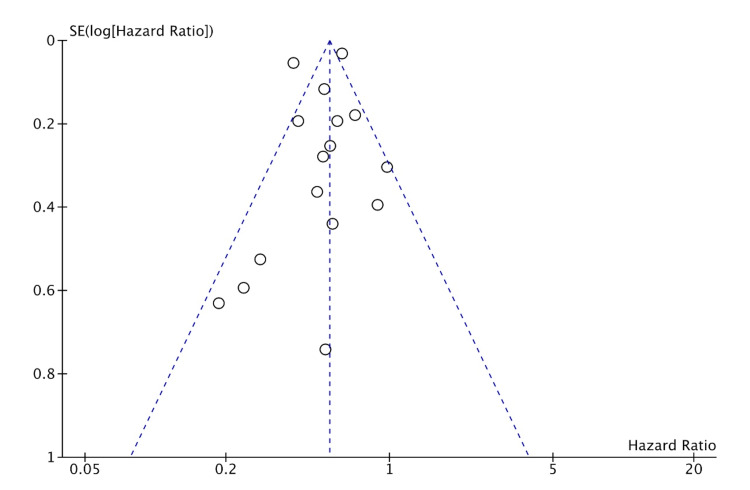
Funnel plot depicting symmetrical distribution for myocardial infarction

**Figure 9:**
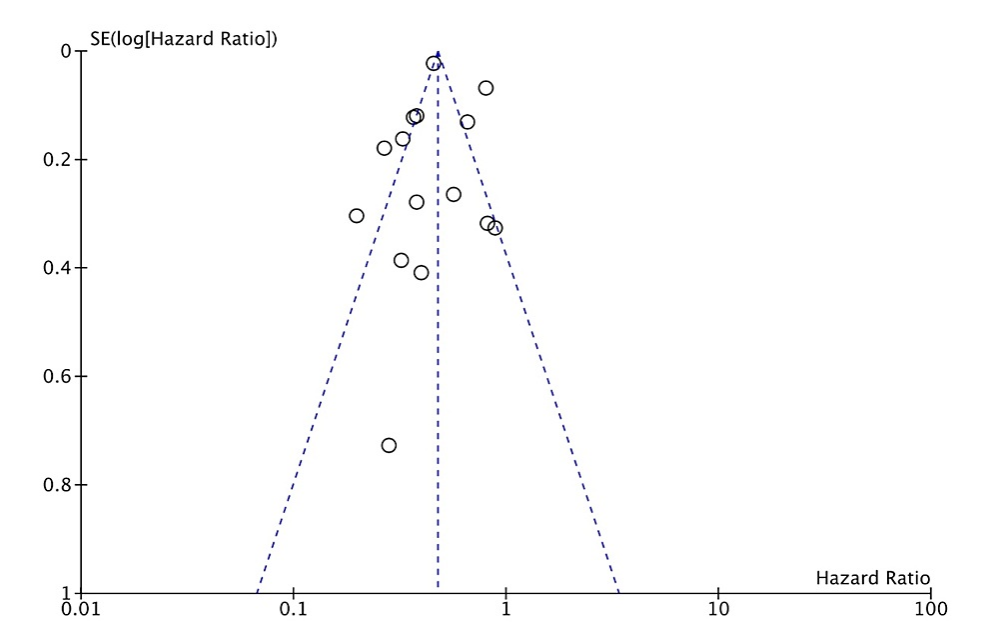
Funnel plot depicting symmetrical distribution for heart failure

**Figure 10:**
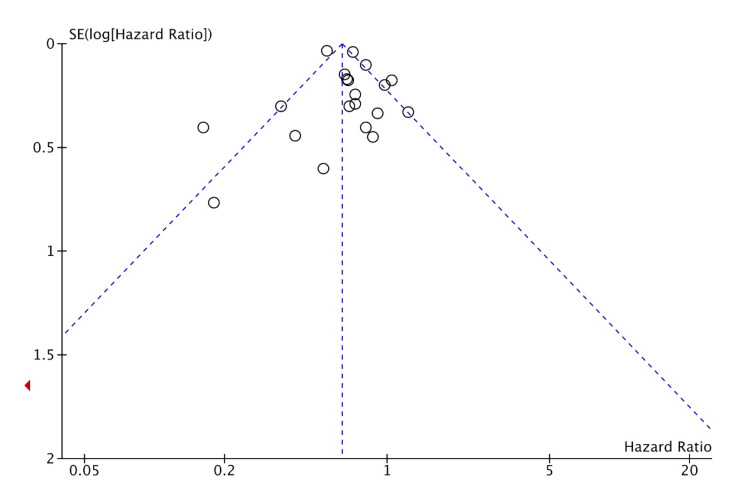
Funnel plot depicting symmetrical distribution for cerebrovascular accident

**Figure 11:**
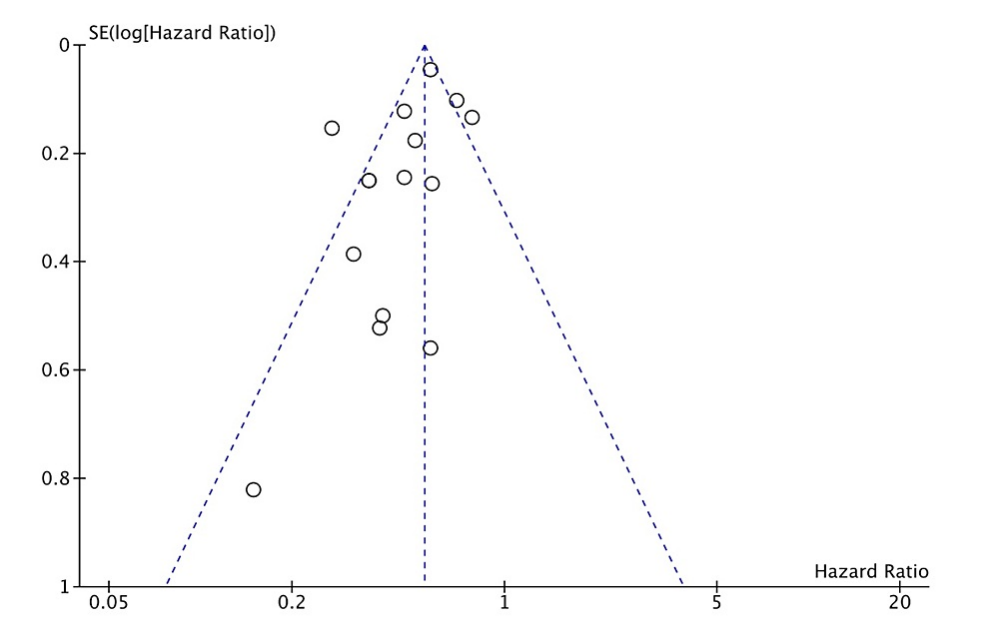
Funnel plot depicting symmetrical distribution for cardiovascular mortality

Publication bias was assessed for MI, HF, CVA, and CVD. The studies included had a moderate-to-high amount of heterogeneity. This is likely from many smaller studies included leading to effect size variation. This is suggestive of likely publication bias in favor of positive studies. But the funnel plots (shown in Figures 8-11) show the studies being symmetrically scattered around the midline. This is in concordance with the inverted funnel appearance reassuring that there is no publication bias [63].

Discussion

In this updated meta-analysis, we analyzed six major long-term cardiovascular outcomes post-bariatric surgery. Five outcomes including CAD, MI, HF, CVA, and CVD mortality showed a significant risk reduction, whereas atrial fibrillation showed a non-significant risk reduction.

Bariatric Surgery and Atherosclerotic Disease

Obesity poses a high risk for atheroma formation [2]. Bariatric surgery provides a beneficial effect by altering molecular mechanisms involving inflammation. Bariatric surgery decreases the levels of oxidative stress and inflammatory markers [64]. It reduces circulating levels of adhesion molecules and improves endothelium-dependent vasodilatory response [65]. Objectively, several studies have shown that surgery reduces carotid intimal wall thickness in concordance with weight loss [66]. These processes in turn contribute to the risk reduction of atherosclerotic diseases such as CAD, MI, and CVA.

Although CAD and MI are atherosclerotic processes, they differ in their pathophysiology and clinical manifestations. CAD is defined as the presence of atherosclerotic plaque within the epicardial coronary arteries. Over time, risk factors potentiate plaque growth. During periods of myocardial oxygen demand, there is endothelial dysfunction causing plaque rupture. This in turn leads to atherothrombosis, vessel occlusion, and myocardial infarction [67]. Of significance, there was a 29.3% cumulative decrease in MI-related inpatient deaths and 3.6% cumulative increase in CAD-related inpatient deaths from 2001 to 2014 [68]. It is important to differentiate MI and CAD, as bariatric surgery is protective against both MI and CAD. Hence, we have studied the effects separately.

The pooled HR for CAD in our study was 0.68 (95% CI: 0.52-0.91). Currently, there are no prior meta-analysis exhibiting the association between bariatric surgery and CAD. The pooled HR for MI in our meta-analysis was 0.53 (95% CI: 0.44-0.64) from 16 studies. This is in concordance with previous studies. Kwok et al. reported a pooled OR of 0.46 (95% CI: 0.30-0.69) from four studies [69]. A more recent analysis by van Veldhuisen et al. reported a pooled HR of 0.58 (95% CI: 0.43-0.76) from seven studies [70]. The pooled HR for composite CVA in our meta-analysis was 0.68 (95% CI: 0.59-0.78) from 21 studies. Kwok et al. reported a similar pooled OR of 0.49 (95% CI: 0.32-0.75) from four studies [69].

Bariatric Surgery and Heart Failure

Bariatric surgery counteracts the effects of obesity on the heart, as described previously. Although there are no randomized controlled trials to show this effect on heart failure, few observational studies have been conducted. The mechanism by which this occurs could be multifactorial. Bariatric surgery reduces heart failure risk factors including hypertension, hyperlipidemia, and diabetes [51]. It also directly acts on the myocardium causing changes in the left ventricle (LV) wall and ejection fraction (EF) percentage. Vest et al. showed that bariatric surgery improved left ventricular systolic dysfunction and resulted in a statistically significant improvement in left ventricle ejection fraction (LVEF) [71]. Another study showed a 43% reduction in left ventricular mass with subsequent reduction in left atrial and right ventricular wall diameter and epicardial fat [72]. A meta-analysis done by Cuspidi et al. showed significant changes in LV thickness, improvement in LV diastolic function, and a decrease in left atrial diameter [73]. Cuspidi et al. also showed no significant improvement of EF percentage [73]. The pooled HR for HF in our study was 0.45 (95% CI: 0.37-0.55) from 15 studies. This is consistent with a prior similar meta-analysis [70,74].

Bariatric Surgery and Cardiovascular Mortality

Scandinavian countries have the most comprehensive obesity registries with a long-term follow-up [14,25,35,43,57]. The data from these have provided significant insight into the long-term outcomes after bariatric surgeries. Carlsson et al. followed 2007 patients over a mean of 24 years and found 457 deaths, of which 167 were from cardiovascular causes, the most common cardiovascular cause of death being myocardial infarction, heart failure, and sudden death [14]. Kauppila et al. reported from the Nordic population. Among 49977 patients that underwent bariatric surgery, there were 525 cardiovascular deaths with patients followed up to >15 years [57]. Sjöström et al. studied 2010 subjects with a mean follow-up of 14.7 years, encountering 28 cardiovascular deaths [25].

In our analysis, the pooled HR for CVD mortality was 0.48 (95% CI: 0.40-0.57) involving 15 studies. Our study has the largest pooled data with respect to cardiovascular mortality data to date. Wiggins et al. reported an OR of 0.50 (95% CI: 0.39-0.71) from three studies [75].

Given the significant cardiovascular benefits offered by bariatric surgery, the referral from primary care physicians has been lower. This could be attributed to knowledge gaps, hesitancy, or concerns regarding postoperative care. A recent Canadian survey showed that more than 50% of physician respondents did not feel equipped to counsel the patients on surgical options. And only 11.6% of the obese patients were being counselled [76]. In a Swedish survey, interestingly, 84% of respondents stated that the patients themselves initiated bariatric surgery referral [77]. Physician’s knowledge showed a positive correlation toward referral and management of postoperative issues [77]. This brings into perspective that education and awareness would lead to better patient sampling, thereby cumulatively improving cardiovascular outcomes.

Limitations

Firstly, the studies included are all nonrandomized cohort studies, which could involve selection and publication biases. Henceforth, longer randomized controlled trials are required. Secondly, most of the outcomes had high heterogeneity, which could be owed to the many smaller studies that were included. Thirdly, some studies had non-generalizable populations such as type 1 diabetes or type 2 diabetes specifically. However, we omitted populations that had cardiovascular diseases at baseline. Fourthly, only English studies were included owing to the ease of interpretation and analysis. Lastly, we failed to study the HR specific to each bariatric surgery, likely due to the scarcity of data for a pooled analysis.

## Conclusions

Although the management of obesity requires a multimodal approach, recognizing the necessity for bariatric surgery early in the disease course is important. Both the physician and the patients should be aware of the treatment strategies to make a well-informed decision. Our study is an updated meta-analysis highlighting the consistency with the prior data. We included additional studies to provide more comprehensive data on six major cardiovascular outcomes. In conclusion, bariatric surgery showed a statistically significant risk reduction with CAD, MI, HF, CVA, and cardiovascular disease-specific mortality and a non-significant risk reduction of atrial fibrillation. However, these data are inclusive of RYGB, SG, and laparoscopic banding. Further research needs to be conducted to determine if these individual procedures have better overall outcomes than one another.​​​​​
